# Single-Cell RNA Sequencing Reveals the Cellular and Molecular Differences Between Myxofibrosarcoma and Undifferentiated Pleomorphic Sarcoma

**DOI:** 10.3390/medsci14010077

**Published:** 2026-02-10

**Authors:** Timur I. Fetisov, Alexander V. Ikonnikov, Elena E. Kopantseva, Polina A. Shtompel, Sofya A. Khazanova, Ekaterina S. Trapeznikova, Victoria Y. Zinovieva, Svetlana N. Zuevskaya, Anastasia A. Tararykova, Beniamin Yu. Bokhyan, Gennady A. Belitsky, Ekaterina A. Lesovaya, Marianna G. Yakubovskaya, Evgeny V. Denisov, Kirill I. Kirsanov

**Affiliations:** 1 Institute of Medicine, RUDN University, 117198 Moscow, Russia; timkatryam@yandex.ru (T.I.F.); y.kopantseva@gmail.com (E.E.K.);; 2 Department of Chemical Carcinogenesis, Institute of Carcinogenesis, N.N. Blokhin National Medical Research Center for Oncology, 115522 Moscow, Russia; 3 Department of Infectious Diseases, Institute of Public Health Named After F.F. Erisman, Sechenov First Moscow State Medical University (Sechenov University), 119048 Moscow, Russia; 4 Oncology Department, Ministry of Health of Russia, Ryazan State Medical University Named After Academician I.P. Pavlov, 390026 Ryazan, Russia

**Keywords:** undifferentiated pleomorphic sarcoma, myxofibrosarcoma, single-cell RNA sequencing, gene expression, intercellular interactions

## Abstract

**Objective:** Myxofibrosarcoma (MXF) and undifferentiated pleomorphic sarcoma (UPS) are common and aggressive subtypes of cancer differing by clinical characteristics and prognosis; however, their differential diagnosis is difficult. Elucidation of cellular and transcriptomic discrepancies between these diseases that could improve their identification was the aim of our study. **Methods:** We applied single-cell RNA sequencing to compare MXF and UPS by tumor cell clusters and cell–cell ligand–receptor interactions, using five tumor samples of both subtypes. **Results:** We identify nine major cell types in all tumors analyzed. Any significant differences in their proportions between MXF and UPS were not found. Further reclusterization of lymphoid cells showed that cytotoxic CD8+ T cell proportion was higher in the MXF samples. In UPS cancer cells, the pathways maintaining extracellular matrix components (including collagens, proteoglycans, and other proteins) were highly active, while MXF cells were characterized by high activity of growth factors and angiogenesis pathways. The ligand–receptor interactions between cancer cells and the microenvironment differed significantly between MXF and UPS. In UPS, CD80 of dendritic cells and macrophages prominently interacted with T cell co-inhibitory CTLA-4 receptors, whereas the activating CD80-CD28 interaction was predominant in MXF. Moreover, in UPS, CD44 and integrins of cytotoxic CD8+ T cells prominently interacted with COL1A1/2, while in MXF CD44, interaction with FN1, COL6A1, and LAMC1 prevailed. **Conclusions:** Differences were identified between UPS and MFS in the composition of lymphoid cell populations and in the intercellular interactions. This proposes deeper understanding of the biological differences between these sarcoma subtypes and may be important for the development of new therapeutic approaches, although further validation of the findings is required.

## 1. Introduction

Among the malignant tumors of soft tissues, undifferentiated pleomorphic sarcomas (UPS) and myxofibrosarcomas (MXF) are characterized by aggressiveness, including a tendency towards recurrence and metastasis [1,2,3]. The type of disease progression that influences the treatment strategy distinguishes these two tumor types. MXF is characterized by the infiltrate growth and increased frequency of local recurrences, while UPS displays increased frequency of distant metastasis [1,4]. However, cell morphology and cell marker expression in these two tumor types bear a high degree of homology, making a correct diagnosis difficult [4]. Although UPS is characterized by the exceptionally deep location of the tumor and medium-to-high malignancy grade, while the malignancy grade of MXF can span from low to high, and their location spans from surface-level to deep inside the layer of tissue, these facts hardly help to make differential diagnosis between UPS and MXF easier [5]. Moreover, according to the TCGA data, UPS and MXF have similar profiles of mutations and DNA methylation, in particular, in the TP53, RB1, CDKN2A, and ATRX gene abnormalities [6]. Based on data from genomic and transcriptomic profiling, some authors even consider these two tumor types as one united tumor group (UPS/MXF) [6,7]. Elucidation of histopathologic features of tumors that could improve differential diagnosis between UPS and MXF remains an important problem for disease prognosis and control, and it requires the development of new approaches.

There is a growing body of data on the immune microenvironment of these two tumor types. Recent investigations demonstrated that the tumor microenvironment (TME) in UPS is characterized by the high presence of myeloid cells [8,9,10]. The comparison of UPS and MXF displays that the CD163+ and CD163+HLA-DR+ myeloid subtypes are more widely represented in UPS than in MXF [10]. Taken together, similarities and differences in the course of the disease, frequency of local and distant metastasis, morphological, mutational, and epigenetic characteristics of tumors, and features of TME indicate the need for further molecular analysis of UPS and MXF using modern, sophisticated techniques. Single-cell RNA sequencing (scRNA-seq) has opened up new opportunities for studies of tumor heterogeneity, including the molecular profiles of single cells and the analysis of interactions between tumor cell populations.

In this study, we performed single-cell RNA sequencing for the comparative analysis of cluster presentation and cell–cell interactions of cancer cells and tumor microenvironment (TME) cells in myxofibrosarcoma (MFS) and undifferentiated pleomorphic sarcoma (UPS). The obtained data broaden our understanding of the biology of these two sarcoma types, highlight differences in the immune response to these tumors, and, given the growing interest in immunotherapy for these tumors, should be taken into account.

## 2. Materials and Methods

### 2.1. Patient and Sample Collection

Samples of MXF (*n* = 5) and UPS (*n* = 5) were obtained after surgical tumor resection from patients being operated at the N.N. Blokhin National Medical Research Center of Oncology (3 men and 7 women, aged 37–87 years). All tumors were localized in the limbs. The diagnosis was established histologically in all cases based on preoperative biopsy and later confirmed by tumor histological analysis (see Table S1 in the Supplementary Materials). Informed consent for the study and the future publication has been obtained from the patient. The study was conducted in accordance with the Declaration of Helsinki and approved by the Ethics Committee of N.N. Blokhin National Medical Research Center of Oncology (27 October 2020; approval number: 2020-42).

### 2.2. Histopathologic Examination

For conventional light microscopy, tumor tissue was fixed in formalin, embedded in paraffin, sliced, and stained with hematoxylin and eosin. All specimens were reviewed by 2 pathologists. The following WHO 5th edition criteria were used for the differential diagnosis between myxofibrosarcomas and undifferentiated pleomorphic sarcomas. Depth of the primary tumor location: for MXF—superficial location (above the superficial fascia or in the hypodermis), for UPS—deep location. Presence of myxoid matrix: in MXF at least focally, absent in UPS. Furthermore, the presence of the following features, characteristic of both tumor types, was taken into account: the presence of pleomorphic cells, a “null” immunophenotype, and the absence of genetic aberrations typical of other types of pleomorphic sarcomas.

### 2.3. Sample Preparation for Single-Cell RNA Sequencing

Tumor samples of 20–25 mg were excised. The fixation and cellular dissociation of the sample were performed according to the Tissue Fixation and Dissociation Protocol for Chromium Fixed RNA Profiling (10× Genomics, Inc., Pleasanton, CA, USA). The cells were counted using an acridine orange/propidium iodide stain buffer (Logos Bioscience, Dongan-gu, Republic of Korea) on the LUNA-FL Dual Fluorescence Cell Counter (Logos Bioscience, Dongan-gu, Republic of Korea). The cell suspension was stored at −80 °C with 100 µL of Enhancer solution (10× Genomics, Inc., Pleasanton, CA, USA) and 275 µL of 50% glycerol. Before the Chromium Fixed RNA Profiling experiment, the cells were thawed, centrifuged, resuspended in 0.5× PBS with 0.02% BSA, and counted again. A total of 8000 cells from each tumor were included in the scRNA-seq analysis.

### 2.4. Single-Cell RNA Library Construction and Sequencing

The scRNA-seq libraries were made according to the Chromium Fixed RNA Protocol (10× Genomics, USA). The GEM particles were generated on the Chromium iX/X instrument (10× Genomics, Inc., Pleasanton, CA, USA). The amplification steps were performed on the RT-PCR machine QuantGene 9600 (Bioer, Hangzhou, China). The scRNA-seq libraries were sequenced on the Genolab M platform (GeneMind, Shenzhen, China) with the following program: 28 cycles for read 1 and 90 cycles for read 2.

### 2.5. Single-Cell RNA Sequencing Quality Control and Data Processing

Demultiplexing of the scRNA-seq data was performed using the Cell Ranger (version 7.1.0, 10× Genomics, Inc., Pleasanton, CA, USA) pipeline. The data were aligned to the GRCh38-2020-A reference genome. Bioinformatic analysis was conducted using the Seurat package (version 5.0.3) [11]. During preprocessing and Quality Control (QC), thresholds for nFeature and nCount were determined individually for each sample (see Table S2 in the Supplementary Materials). A maximum mitochondrial gene expression content of 10% was applied, and genes expressed in fewer than three cells were excluded. The doublet detection was performed using the DoubletCollection package [12]. The SCTransform method was applied to the expression matrices to carry out data normalization.

### 2.6. Analysis of Differentially Expressed Genes

The filtered cells were grouped into clusters using the Leiden algorithm from the Seurat package. Differentially expressed genes (DEGs) were identified using the FindMarkers function in Seurat with the Wilcoxon signed-rank test and applying the thresholds of log fold change > 1 and adjusted *p*-value < 0.05, with multiple testing correction by the Benjamini–Hochberg method, and requiring expression in at least 50% of the cells within a cluster. The resulting DEGs were used for cluster annotation and identification of function-specific subpopulations within the clusters. Pathways and biological processes enrichment analysis was conducted using EnrichR [13]. Pathways and processes with adjusted *p*-value < 0.05 were considered significantly enriched.

### 2.7. Copy Number Karyotyping of Aneuploid Cells

Copy number alterations were detected using the SCEVAN package [14]. T cells were used as the reference population, and cells classified by the algorithm as aneuploid were considered cancer cells.

### 2.8. Cell–Cell Interaction Analysis

Ligand–receptor interactions between cells were analyzed using the CellChat algorithm [15]. Ligand–receptor interactions were considered significant if the corresponding ligand or receptor met the following over-expression criteria in CellChat: adjusted *p* < 0.05, log 2-fold change > 0.25, and detected in >25% of cells in the cluster.

## 3. Results

### 3.1. Identification of Main Cell Types in UPS and MXF

**Figure 1:**
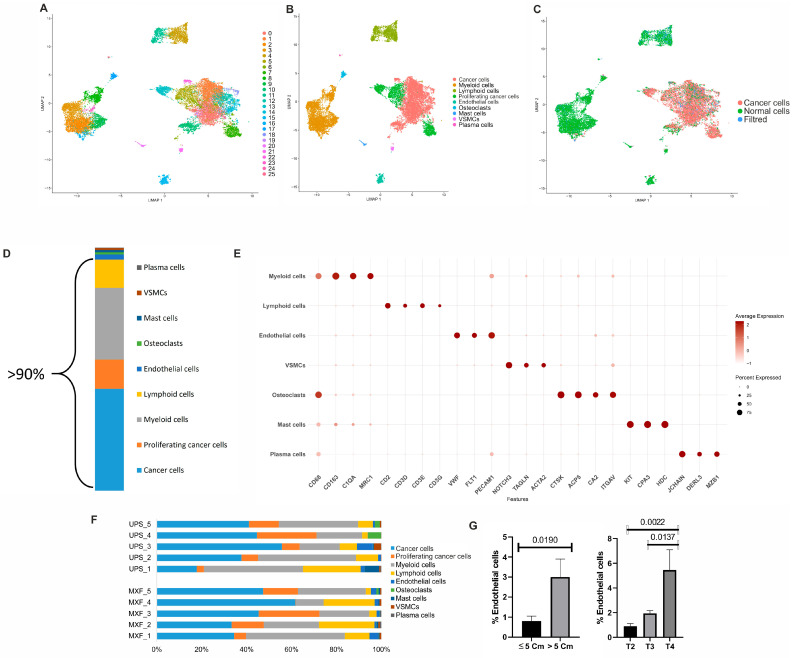
Single-cell RNA-seq profiling of UPS and MXF. (**A**) UMAP of 26 annotated cell clusters; (**B**) UMAP of nine annotated cellular subfamilies; (**C**) UMAP depicting the results of SCEVAN copy number variation prediction. Blue (NA) values indicate that the cell did not have a large enough transcriptome to be evaluated using CopyKAT; (**D**) Major cellular subfamilies; (**E**) Dotplot of the expression levels of classic cell markers in annotated cellular subfamilies. (**F**) Relative proportions of annotated cellular subfamilies. (**G**) The relationships between cellular subfamilies and tumor size (described above).

After quality control and removal of doublets, individual transcriptomes of 26,315 cells were acquired, including 8770 cells from UPS samples and 17,545 cells from MXF samples. The clusterization according to the Leiden method led to the detection of 26 cell clusters. Based on the results of aneuploidy analysis with the SCEVAN package and the analysis of classic cell markers, these clusters were united into nine main cellular subfamilies of tumor samples: cancer and proliferating cancer cells (aneuploid cells), myeloid cells (*C1QA*, *MRC1*, *CD163*), lymphoid cells (*TRAC*, *CD2*, *TRBC2*, *CD3E-G*), endothelial cells (*PECAM1*, *EGFL7*, *FLT1*, *VWF*), osteoclasts (*CD68*, *CTSK*, *ACP5*, *CA2*), mast cells (*CPA3*, *HDC*, *KIT*), vascular smooth muscle cells (VSMCs: *TAGLN*, *NOTCH3*, *ACTA2*), and plasma cells (*DERL3*, *JCHAIN*, *MZB1*) (Figure 1A–F, Tables S3 and S4). We did not find any significant differences between UPS and MFS in the proportions of the cells of these nine cellular subfamilies, which were rather variable. The cancer cells constituted the majority of cells in both MXF and UPS samples. The prevailing TME cell populations in both sarcoma types were myeloid and lymphoid cells. The analysis of the associations between the relative proportions of the nine annotated cellular subfamilies and the clinical characteristics revealed that endothelial cells are more abundant in tumors over 5 cm in size compared to smaller tumors, and their amount increases with the increase in the tumor size (from T2 to T4 TNM classification) (Figure 1G).

Taking into account that the cancer, proliferating cancer, lymphoid, and myeloid cells constitute more than 90% of cells in the analyzed MXF and UPS samples, these populations were taken for further reclusterization and subsequent analysis (Figure 1D).

### 3.2. Differences in MXF and UPS Gene Expression Profile

**Figure 2:**
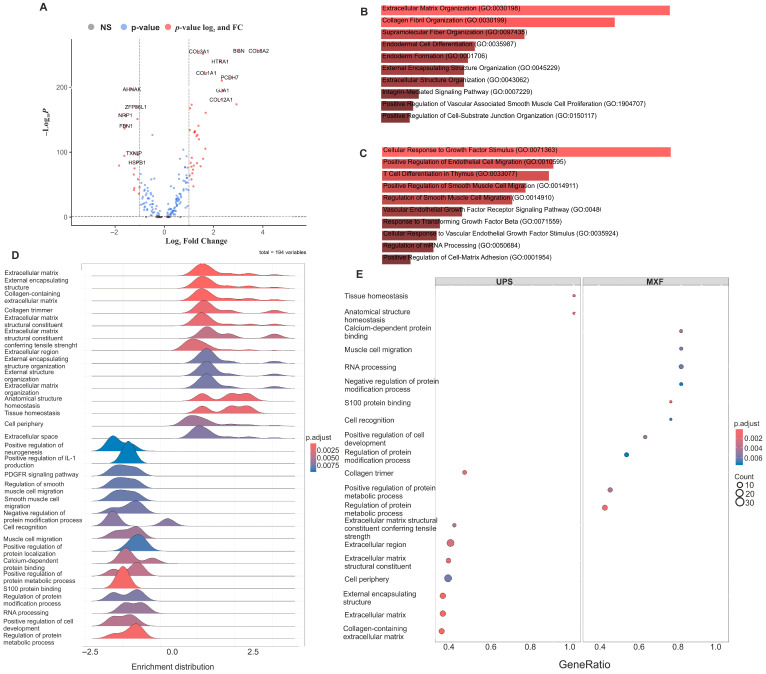
Differences in gene expression profile between MXF and UPS cancer cells. (**A**) Heatmap showing the DEGs between aneuploid cells in MXF and UPS; (**B**) bar graph of major biological process in UPS (GO Biological Process 2025), adjusted *p* value < 0.05; (**C**) bar graph of major biological process in MXF (GO Biological Process 2025), adjusted *p* value < 0.05; (**D**) Gene Set Enrichment Analysis (GSEA) ridge plot; (**E**) GSEA dotplot.

Using the analysis of DEGs for the prevailing cell clusters in MXF and UPS (aneuploid, myeloid, and lymphoid cells), we revealed that the transcriptional profiles of lymphoid and myeloid cell populations were practically identical between MXF and UPS samples, while the transcriptional profiles of aneuploid cells displayed key differences (Figure 2A). The cancer cells in UPS had an enrichment of signaling pathways related to extracellular matrix (ECM) formation, and synthesis and organization of collagen fibrils, while the MXF cancer cells had an enrichment in pathways associated with cell migration, including endothelial and VSMC migration, and cellular response to VEGF and TGF-beta growth factors (Figure 2B–E).

### 3.3. Heterogeneity of Cancer Cells in MXF and UPS

**Figure 3:**
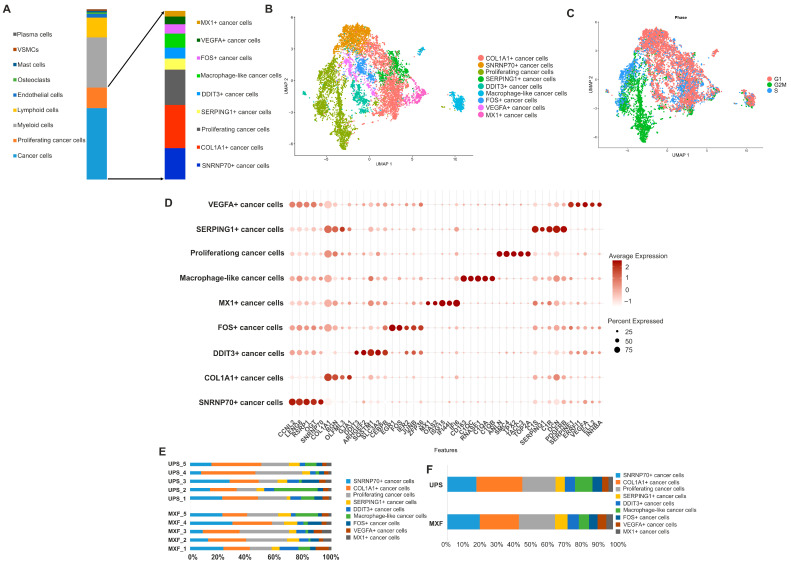
Cancer cell clusters in UPS and MXF. (**A**) Reclustering cancer and proliferating cancer cellular subfamilies; (**B**) UMAP representation of cancer cell clusters; (**C**) UMAP of cell-cycle phase analysis; (**D**) dotplot of the differences in gene expression; (**E**) CC proportions across samples; (**F**) relative CC ratio in UPS and MXF.

Subsequent subcluster analysis was performed for aneuploid cells, including cancer and proliferating cancer cellular subfamilies of all UPS and MXF samples. These cells mainly exhibited high expression of collagens, ECM-remodeling enzymes (*MMP2*, *MMP14*, *LOXL1/2*), and proteoglycans (*DCN*, *BGN*, *LUM*, and *SPARC*). GO, Reactome, and KEGG pathway analysis revealed significant enrichment in pathways associated with the formation and remodeling of ECM, PI3K-Akt, Met, PDGF, and IGF signaling pathways. Seven cancer cell (CC) clusters were identified, including SNRNP70+, COL1A2+, SERPING1+, DDIT3+, FOS+, VEGFA+, MX1+, as well as proliferating CC and the macrophage-like CC clusters (Figure 3A,B). MXF and UPS predominantly contained SNRNP70+, COL1A2+, and proliferating CC, as well as SERPING1+, DDIT3+, FOS+, VEGFA+, MX1+, and the macrophage-like CC minor clusters. The COL1A1+ CC had increased expression of ECM genes (*COL1A1*, *BGN*, *OLFML3*). The SNRNP70+ CC demonstrated the elevated gene expression and signaling pathways associated with RNA splicing (*SNRNP70*, *RSRP1*, *LUC7L*). The proliferating CC showed the enhanced expression of cell proliferation genes (*TOP2A*, *CDK1*, *KIF23*, *NUSAP1*) and pathways. This CC was also significantly enriched in the S and G2/M phases of the cell cycle, compared to other cancer clusters (Figure 3C and Figure S1). Among minor CC, the SERPING+ CC was distinguished by the enrichment of ECM remodeling (*HTRA3*, *CTSK*, *MFAP4*, *DCN*) and complement system (*C1R*, *C1S*, *SERPING1*, *SERPINF1*) genes and signaling pathways. Another minor DDIT3+ CC demonstrated the upregulation of genes involved in cellular stress response (*DDIT3*, *ARHGEF2*, *SLC3A2*) and the corresponding pathways. The FOS+ CC demonstrated the overexpression of oncogenes (*FOS*, *EGR1*, *IER2*) and a considerable enrichment in NGF-stimulated transcription, as well as positive regulation of miRNA transcription and the IL-17 signaling pathway. The VEGFA+ CC displayed high expression of the VEGFA, ERRFI1, and INHBA genes involved in the regulation of angiogenesis and cellular response to hypoxia, as well as enrichment in dissolution of fibrin clot, PI3K-Akt, and vascular endothelial growth factor signaling pathways. The MX1+ CC was distinguished by the enrichment of MX1, ISG15, and OAS2 genes and pathways involved in interferon signaling (Figure 3D, Supplementary Files). Additionally, we revealed a CC with increased expression of macrophage-specific markers, such as CD163 and CD68, complement system genes (*C1QC*, *C1QB*, *C1QA*), VSIG4, and cathepsins (*CTSB*, *CTSZ*, *CTSC*, *CTSD*). The proportions of CC clusters varied both in UPS and in MXF (Figure 3D,E, and Table S4).

### 3.4. Heterogeneity of Myeloid Cells in MXF and UPS

**Figure 4:**
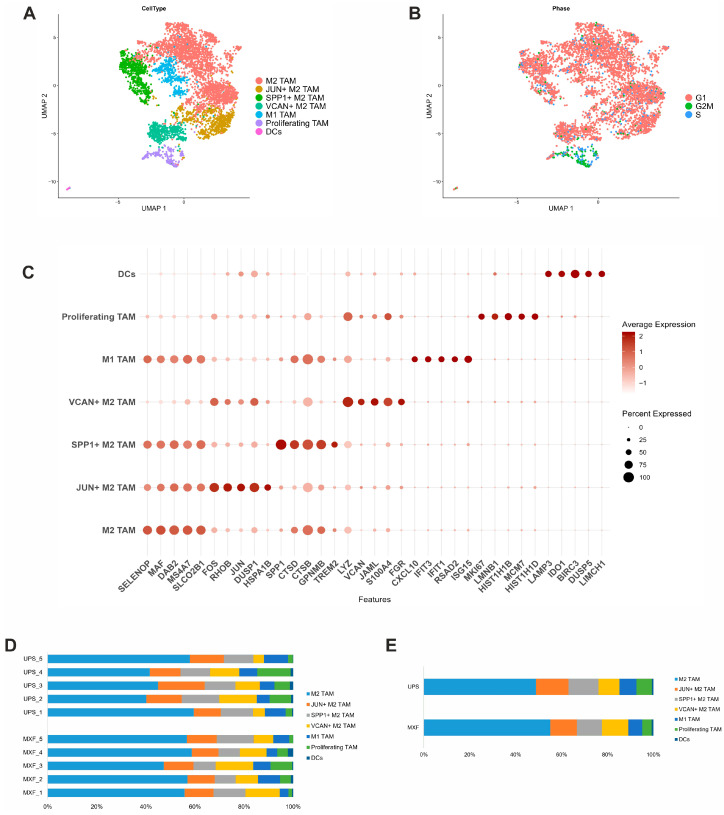
Myeloid cell clusters in UPS and MXF. (**A**) UMAP representation of myeloid cell clusters; (**B**) UMAP of cell-cycle phase analysis; (**C**) dotplot of the differences in gene expression; (**D**) myeloid cluster proportions across samples; (**E**) relative myeloid cluster ratio in UPS and MXF.

Myeloid cells both in UPS and MXF samples were marked by the high expression of the MS4A (MS4A4A, MS4A7), CD68, and CD163 genes, as well as enrichment in the innate immune system, lysosome, and receptor-mediated endocytosis. After reclusterization, the myeloid cells were distributed among the seven clusters: four clusters of M2 macrophage, one cluster of M1 macrophage, one cluster of proliferating myeloid cells, and one cluster of dendritic cells (Figure 4A,B). The first M2 macrophage cluster with increased expression of classic M2 markers (CD68, CD163) and selenoprotein P (SELENOP) genes was predominant in both MXF and UPS. The second JUN+ M2 macrophage cluster expressed the heat shock protein genes (HSPA1A and HSPA1B) and oncogenes (JUN, FOS, and JUND). Moreover, AP-1, non-receptor tyrosine kinases, and apoptosis signaling pathways were significantly enriched in this cluster. The third SPP+ M2 macrophage cluster was enriched in genes involved in the degradation of the extracellular matrix, lysosome, and antigen processing, as well as the presentation of exogenous peptide antigen via MHC class II. The fourth VCAN+ M2 macrophage cluster displayed high expression of the VCAN, S100A9, and CLEC10A genes, as well as genes involved in the differentiation of myeloid cells and leukocyte adhesion. The M1 macrophage cluster was characterized by the upregulation of genes typical for the M1 phenotype (CXCL10, IFIT1, IFIT3) and an enrichment in the interferon signaling pathway. The proliferating myeloid cluster displayed the highest proportion of cells in the S and G2/M phases of the cell cycle and increased expression of the MKI67 gene (Figure 4C). The dendritic cell cluster displayed increased expression of dendritic cell markers (LAMP3, LY75, CD83) and an enrichment in interleukin-2 family signaling, NF-kappa B, and TNF signaling pathways (Figure 4C and Figure S2). The proportions of cells in different myeloid clusters varied in both UPS and MXF (Figure 4D,E and Table S4).

### 3.5. Heterogeneity of Lymphoid Cells in MXF and UPS

**Figure 5:**
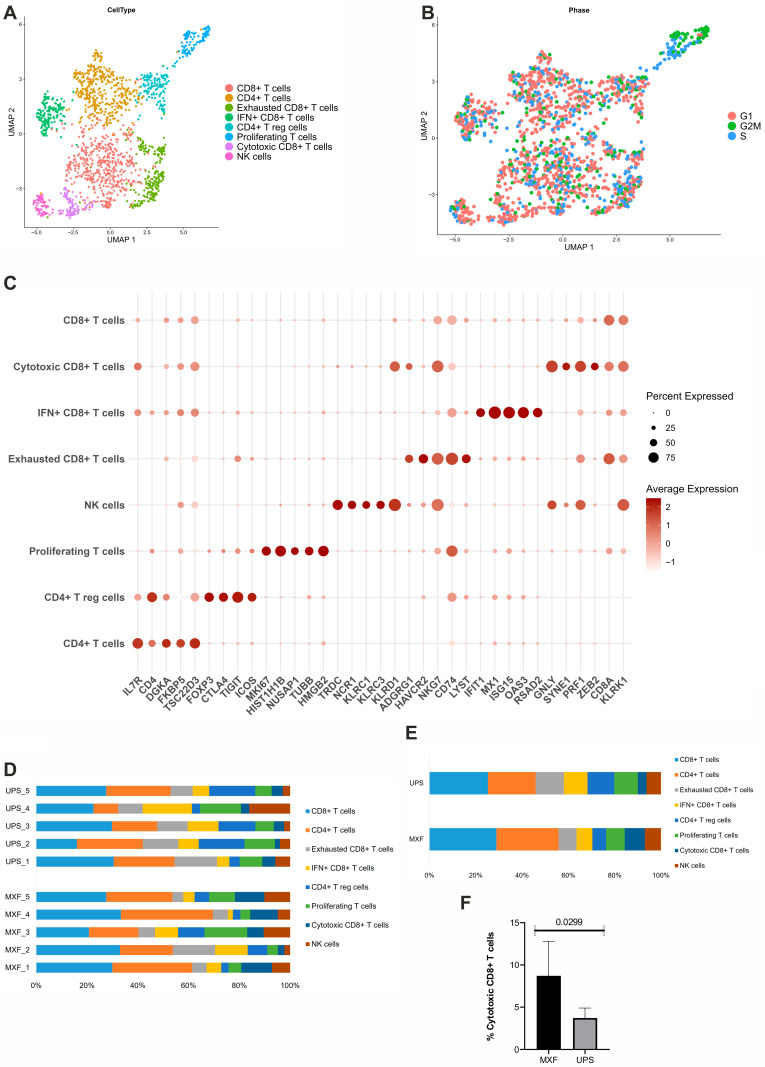
Lymphoid cell clusters in UPS and MXF. (**A**) UMAP representation of lymphoid cell clusters; (**B**) UMAP of cell-cycle phase analysis; (**C**) dotplot of the differences in gene expression; (**D**) lymphoid cluster proportions across samples; (**E**) relative lymphoid cluster ratio in UPS and MXF; (**F**) cytotoxic CD8+ T cell cluster prevalence in the UPS and MXF.

Lymphoid cells in both MXF and UPS were marked by the increased expression of markers of T and NK cells (*CD3D*, *CD2*, *CD96*, *CD3E*) and an enrichment in the immune system, primary immunodeficiency, and T-cell receptor signaling pathways. After reclusterization, the lymphoid cells were distributed among eight clusters: four clusters of CD8+ T cells (CD8+ T cluster, exhausted CD8+ T cells, IFN+ CD8+ T cells, cytotoxic CD8+ T cells), two clusters of CD4+ T cells including CD4+ T cells and CD4+ Treg cells, a cluster of proliferating T cells, and a cluster of NK cells (Figure 5A,B). The prevailing lymphoid clusters in both MXF and UPS were the CD8+ and CD4+ T cells. The CD8+ T cells showed overexpression of the KLRK1 gene, which is necessary for their cytotoxic activation. The CD4+ T cells were characterized by the upregulation of the IL7R gene. The ADGRG1+ CD8+ T cell cluster had high expression of genes associated with activation of T cells (*ADGRG1*, *LYST*, *NKG7*, *CD27*) and exhaustion markers, such as HAVCR2 and LAG3. The IFIT+ CD8+ T cell cluster was enriched by the interferon (*IFIT1*, *RSAD2*, *ISG15*) genes and signaling pathways. Also, a cell cluster with increased expression of genes typical for T reg cells (*FOXP3*, *CTLA4*, *TIGIT*) was identified. This cluster was enriched with genes associated with RUNX1 and FOXP3, which control the development of regulatory T lymphocytes, PD-L1 expression, and the PD-1 checkpoint pathway in cancer, as well as the regulation of the canonical NF-kappaB signal transduction pathway. The GNLY+ cluster demonstrated expression of CD8A, the overexpression of genes associated with cytotoxic activity of T cells and NK cells (*GNLY*, *KLRG1*, *GZMH*, *NKG7*, *EFHD2*), and an enrichment in the immune system, antigen processing and presentation, natural killer cell-mediated cytotoxicity, and cellular response to lectin pathways (Figure 5C and Figure S3). Considering this data, the GNLY+ cluster was labeled as the cytotoxic CD8+ T cell cluster. It is important to note that in the UPS samples, the representation of the cytotoxic CD8+ T cell cluster was significantly lower than in the MXF (*p* < 0.05; Figure 5F). We also isolated the cluster of proliferating T cells, which showed the high expression of proliferation genes (*HIST1H1B*, *MKI67*, *NUSAP1*, *HIST1H1C*) and an enrichment in the DNA geometric change (GO:0032392), mitotic chromosome condensation (GO:0007076), gene and protein expression by JAK-STAT signaling after interleukin-12 stimulation, and Rap1 signaling pathways. Furthermore, the analysis of cell distribution among cell cycle phases confirmed the high proliferative activity of this lymphoid cluster (Figure 5C). The NCR1+ cluster expressed markers of NK cells (*FCGR3A* and *NCAM1*) and genes associated with cytotoxic activity of T and NK cells (*NCR1*, *KLRC1*, *KLRC3*) (Figure 5D,E, and Table S4).

### 3.6. The Differences in Cell–Cell Interactions Between UPS and MXF

**Figure 6:**
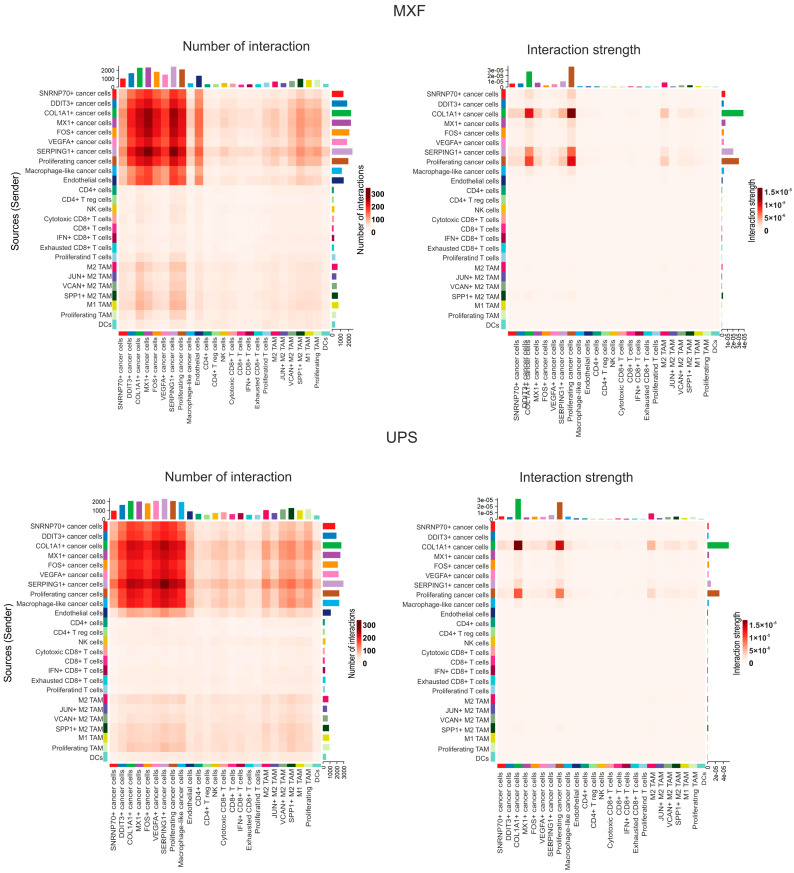
Cell–cell interactions in UPS and MXF. Heatmap depicting the overall number of interactions and the strength of interactions for MXF and UPS.

Here, we performed an analysis of intercellular communication, focusing on the main clusters of UPS and MXF: aneuploid, myeloid, and lymphoid. Furthermore, given the high interest in anti-angiogenic drugs for soft tissue sarcoma therapy, the endothelial cell cluster was also included in the analysis [16]. In both tumor types, the greatest number of interactions involved aneuploid, myeloid, and endothelial cells. Notably, in MXF, cancer cells interacted more actively with endothelial cells compared to UPS (Figure 6). The strongest interactions in UPS were observed between the proliferating and COL1A1+ aneuploid cell clusters, whereas in MXF, the strongest interactions were between the proliferating, COL1A1+, and SERPING1+ aneuploid cell clusters (Figure 6).

**Figure 7:**
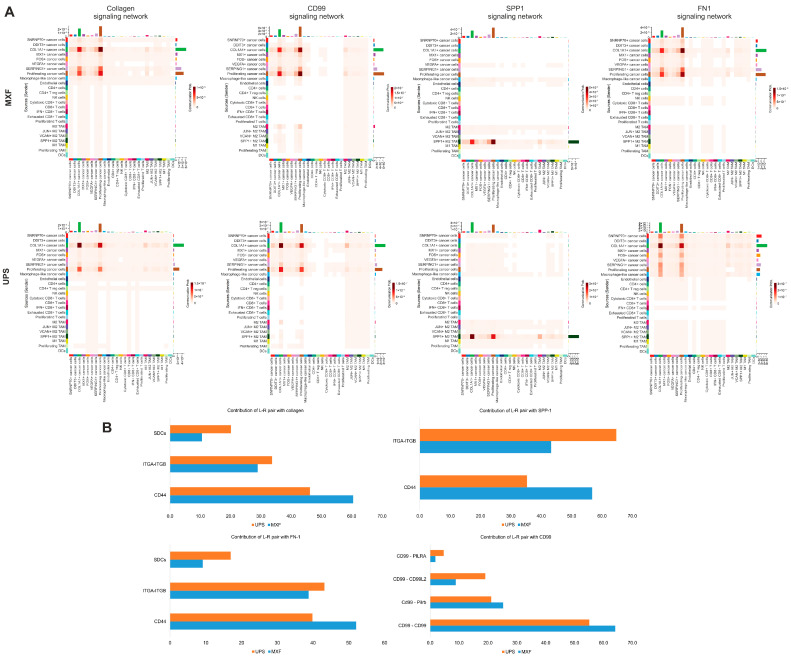
Cell–cell interactions in UPS and MXF. (**A**) The most pronounced interactions; (**B**) ligand–receptor contributions between cells.

For both tumor types, the most likely signaling pathways are collagen, CD99, FN1, and SPP1. Upon detailed examination, differences were identified in specific ligand–receptor pairs within the collagen, CD99, FN1, and SPP1 signaling pathways. For MXF, the largest number of probable interactions of collagens FN1 and SPP1 were with CD44, whereas for UPS, interactions of these ligands were more evenly distributed between integrins, CD44, and syndecans. Furthermore, interactions of CD99 with CD99L2 and PILRA were probable in UPS (Figure 7A,B).

**Figure 8:**
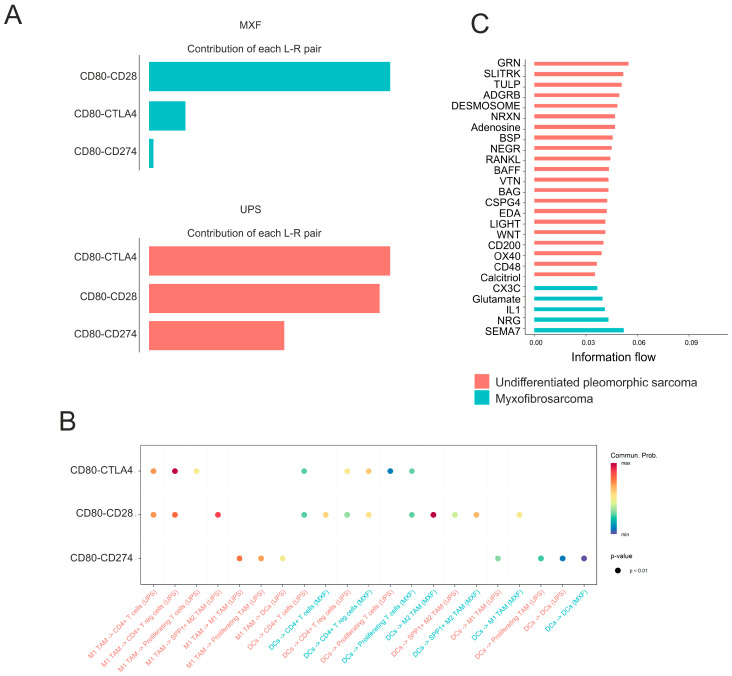
Cell–cell interactions in UPS and MXF. (**A**) Heatmap depicting the overall number of interactions and strength of interactions for MXF and UPS; (**B**) the most pronounced interactions; (**C**) ligand–receptor contributions between cells.

Given our finding of a difference in the abundance of the cytotoxic CD8+ T cell population in UPS and MXF, we analyzed cell–cell interactions between these and other cells. It was shown that in UPS, cytotoxic CD8+ T cells most likely interact with all cancer clusters via Collagen 1 and Collagen 6 with CD44 and ITGA1/ITGB1, as well as with SPP1+ M2 tumor-associated macrophages (TAM) via the SPP1 ligand. In contrast, in MXF, cytotoxic CD8+ T cells interact only with COL1A1+, SERPING1+, proliferating, and MX1+ cancer cells via FN1, COL6A1, and LAMC1 ligands with CD44 (Figure 8A).

Considering the growing interest in immunotherapy for UPS and MXF, the activity of CD80 interactions with CD28, CTLA4, and CD274 ligands was analyzed in the studied sarcoma types. It was revealed that the CD80-CD28 interaction between dendritic cells and M2 macrophages was most probable in MXF (Figure 8B). For UPS, interactions of CD80 with both CTLA-4/CD274 and CD28 were probable. The main donor was the M1 macrophage cluster, which interacted with T cells (CD4+ T cells and Treg cells), and dendritic cells interacted with these cells (Figure 8B).

To further examine overall differences in information flow in cellular communication in UPS and MXF, we used the sum of communication probabilities between cell population interaction pairs to infer cellular information flow in the network [17]. We identified 21 signaling pathways unique to UPS, 11 of which originated from cancer cells, including 5 interactions with other cancer cells (SLITRK, ADGRB, NRXN, NEGR, CSPG4) and 6 pathways with microenvironment cells (GRN, Adenosine, BSP, RANKL, BAG, TULP). Macrophages acted as ligand donors in desmosome, BAFF, and EDA signaling pathways with cancer cells. Dendritic cells also interacted with cancer cells via VTN and calcitriol. NK and lymphoid cells were donors in LIGHT, CD48, and OX40 pathways, and endothelial cells interacted with macrophages via CD200 (Figure 8C and Figure S4). Additionally, five unique signaling pathways were identified for MXF, such as SEMA7 and glutamate between cancer cells, IL18 and NRG between macrophages and NK/cancer cells, and CX3C between endothelial cells and lymphoid cells (Figure 8C and Figure S4).

## 4. Discussion

This study presents the first single-cell RNA sequencing data of two related sarcoma types, myxofibrosarcoma (MXF, *n* = 5) and undifferentiated pleomorphic sarcoma (UPS, *n* = 5), along with their comparative analysis. Both tumor types exhibited a highly heterogeneous cellular composition. We described nine cell populations common to both, with the main cellular landscape of both UPS and MXF predominantly consisting of four cell types: cancer and proliferating cancer, myeloid, and lymphoid cells. Overall, the cellular composition of UPS and MXF was comparable.

The tumors studied differed significantly in the abundance of cytotoxic CD8+ T cells. These cells, along with NK cells, are the most potent effectors of the anti-tumor immune response and form the basis of modern cancer immunotherapies [18]. An increased abundance of cytotoxic CD8+ T cells is associated with a favorable response to immune checkpoint inhibitors (ICI) [19]. Therefore, the difference in the abundance of these cells may underlie the differential response to immunotherapy between UPS and MXF.

Despite similar clusters, cancer cells in UPS showed higher expression of collagens and their modifying enzymes, whereas MXF exhibited higher expression of genes regulating endothelial cell migration and responses to growth factor signaling, including VEGF and TGF-beta. Our findings complement the results of Pan et al., who demonstrated higher expression of the collagen-modifying enzyme Plod2 in UPS compared to MXF [20]. In our study, we observed upregulation of COL1A1, COL3A1, COL8A2, and COL12A1 in UPS compared to MXF. Overexpression of these genes is known to occur in many cancers and is associated with tumor progression [21,22,23]. Furthermore, accumulating evidence links high expression of COL1A1, COL3A1, and COL12A1 to an immunosuppressive microenvironment, including a reduced abundance of CD8+ cells [24,25,26,27]. This is particularly interesting in light of our data showing interactions between cancer cells of UPS/MXF and cytotoxic CD8+ T cells via COL1 ligand–receptor interactions with CD44 and integrins. Thus, the increased expression of COL1A1 in UPS compared to MXF may explain the lower abundance of cytotoxic CD8+ T cells in UPS.

In contrast to UPS, cancer cells in MXF showed high expression of C1R. Several studies have shown that cancer cell expression of this gene is associated with cell migration and infiltrative tumor growth [28,29], which may explain the greater propensity of MXF for infiltrative growth and local recurrence [1,4]. Additionally, MXF exhibited higher expression of angiogenesis-related genes and the gene for neuropilin-1 (NRP1). This indicates a potential sensitivity of these tumors to anti-angiogenic therapy; however, high NRP1 expression may reduce the efficacy of anti-VEGF agents alone [30].

The analysis of cell–cell interactions revealed that in MXF, the primary role in key interactions, such as those involving collagens, FN1, and SPP1, is attributed to binding with CD44. In contrast, in UPS, besides CD44, these ligands actively interact with integrins and syndecans. Previous studies have demonstrated that high CD44 expression is associated with poor event-free survival and local recurrence in MXF [31]. This high dependency of MXF on CD44 makes it a promising target for therapy, whereas in UPS, inhibiting CD44 would likely lead to compensatory activation of integrins and syndecans.

Furthermore, we identified significant differences in the interactions of CD80 with the activating receptor CD28 and the inhibitory receptor CTLA-4. In MXF, the CD80-CD28 interaction between dendritic cells and M2 macrophages predominated. The functional role of the CD28 signaling pathway in myeloid cells remains a subject of debate; however, accumulating evidence suggests that this interaction leads to the activation of the latter and, consequently, supports a pro-tumor phenotype [32]. In UPS, however, the primary interaction was CD80 with CTLA-4 between M1 macrophages and T cells, leading to T cell inactivation [33]. This difference in interactions could be crucial for understanding the mechanisms of sensitization to immune drugs, highlighting the importance of distinguishing between MXF and UPS.

Beyond the described differences in the microenvironment, we identified pathways characteristic of, or significantly more active in, either UPS or MXF. For UPS, unique immune suppression pathways such as GRN, adenosine, and CD200 are of particular interest [34,35,36]. Additionally, the PD-L1 and PDL2 pathways, involved in immune suppression, were more active in UPS than in MXF. Conversely, in MXF, growth factor pathways such as FGF, HGF, PDGF, and VEGF were more active.

Clinical trials of immune checkpoint inhibitors (ICI) in soft tissue sarcomas are actively underway, with demonstrated anti-tumor efficacy in patients with UPS and MXF [37,38,39]. Therefore, our findings on differential cellular communications between UPS and MXF, especially regarding alternative immunosuppressive pathways in UPS, are highly relevant for applying this therapeutic approach. It was recently shown that inhibiting the GRN pathway in combination with PD-L1 blockade significantly enhances the anti-tumor effect of immunotherapy [40]. We also observed high activity of the OX40 T-cell costimulation pathway, whose agonism also improves tumor response to ICI [41]. Meanwhile, the high activity of growth factor pathways and interactions with endothelial cells in MXF make a combined approach of ICI with tyrosine kinase inhibitors and anti-angiogenic therapy promising for MXF. A recent clinical case report of combined ICI and anti-angiogenic therapy in an MXF patient supports the feasibility of this approach [42,43].

However, the data presented have several limitations. The study analyzed relatively small patient cohorts. Moreover, the data analysis methods themselves, such as copy number variation and cell–cell interaction analysis, despite being widely accepted, are not direct experimental measurements of these parameters but rather computational predictions based on the available data. This highlights the necessity for further validation of the identified patterns, as well as careful extrapolation of the findings for potential clinical application.

In conclusion, this study demonstrates for the first time the distinct differences between UPS and MXF at the transcriptomic level of cell clusters. Although both tumor types were characterized by high immune infiltration and similar mutational profiles, the UPS had a significantly lower abundance of cytotoxic CD8+ T cells compared to MXF. Furthermore, substantial differences were observed in the direction and strength of cell–cell interactions. Overall, our study provides important insights into the single-cell transcriptional profiles of rare sarcomas and highlights key differences between UPS and MFS, laying the groundwork for future research aimed at developing new therapeutic approaches, including immunotherapy.

## Data Availability

The original contributions presented in this study are included in the article/Supplementary Materials. Further inquiries can be directed to the corresponding author.

## Supplementary Materials

The following supporting information can be downloaded at: https://www.mdpi.com/article/10.3390/medsci14010077/s1. Figure S1: Cancer cell cluster signaling pathways; Figure S2: Myeloid cell clusters signaling pathways; Figure S3: Lymphoid cell clusters signaling pathways; Figure S4: Pathway comparison; Table S1: Patient data; Table S2: QC metrics after filtration; Table S3: All cells Manual annot filtered DEGs list (pct 0.5, LFC 1); Table S4: Cell clusters.
