# Supplementary figures and images for: Single-Cell RNA Sequencing Reveals the Cellular and Molecular Differences Between Myxofibrosarcoma and Undifferentiated Pleomorphic Sarcoma
Source: Med Sci (Basel). 2026 Feb 10;14(1):77. doi: 10.3390/medsci14010077 (PMC12922028; doi:10.3390/medsci14010077)

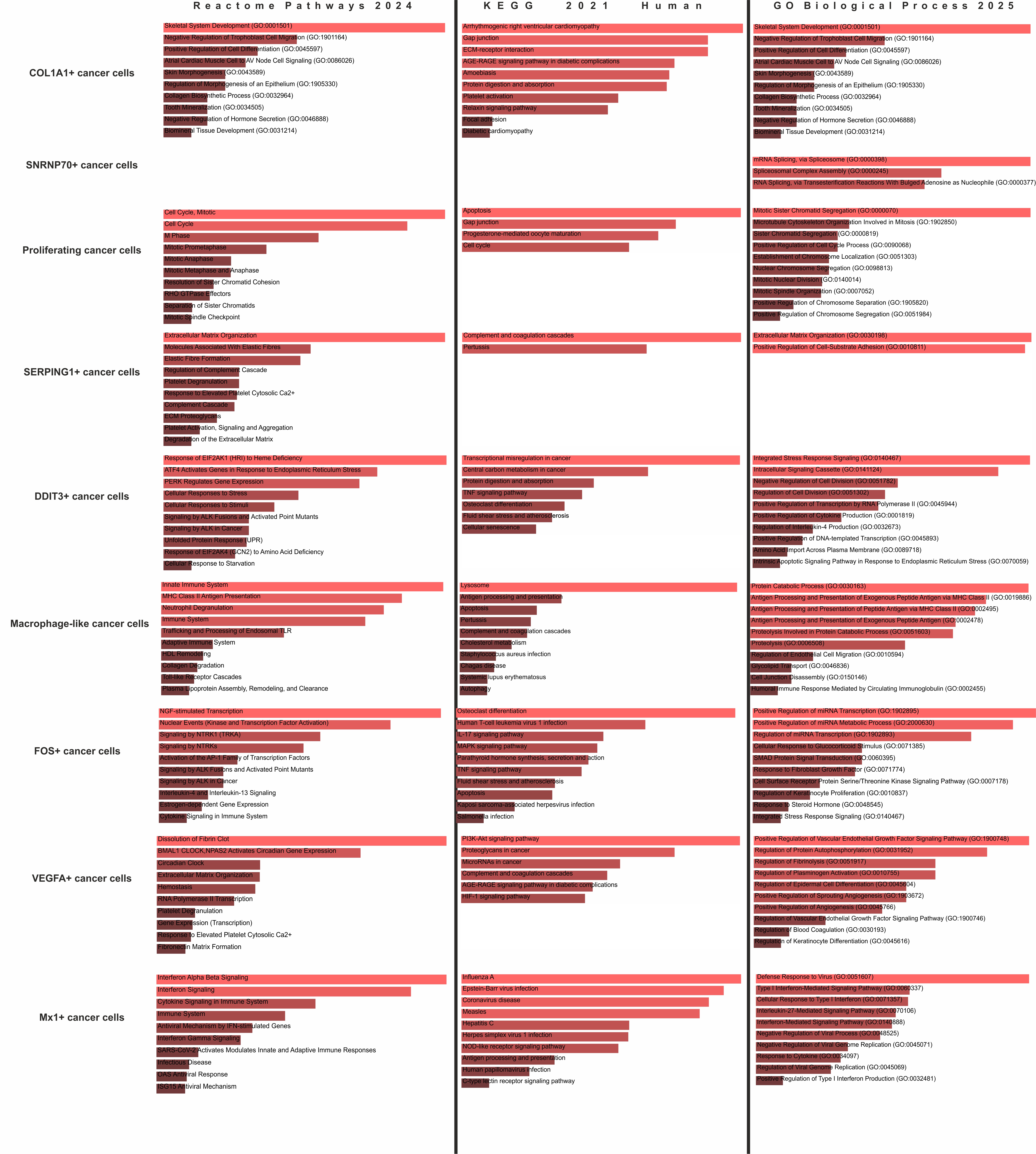

Supplement: Supplementary file 1 [file medsci-14-00077-s001.zip › Figure S1_Cancer cell clusters.jpg]

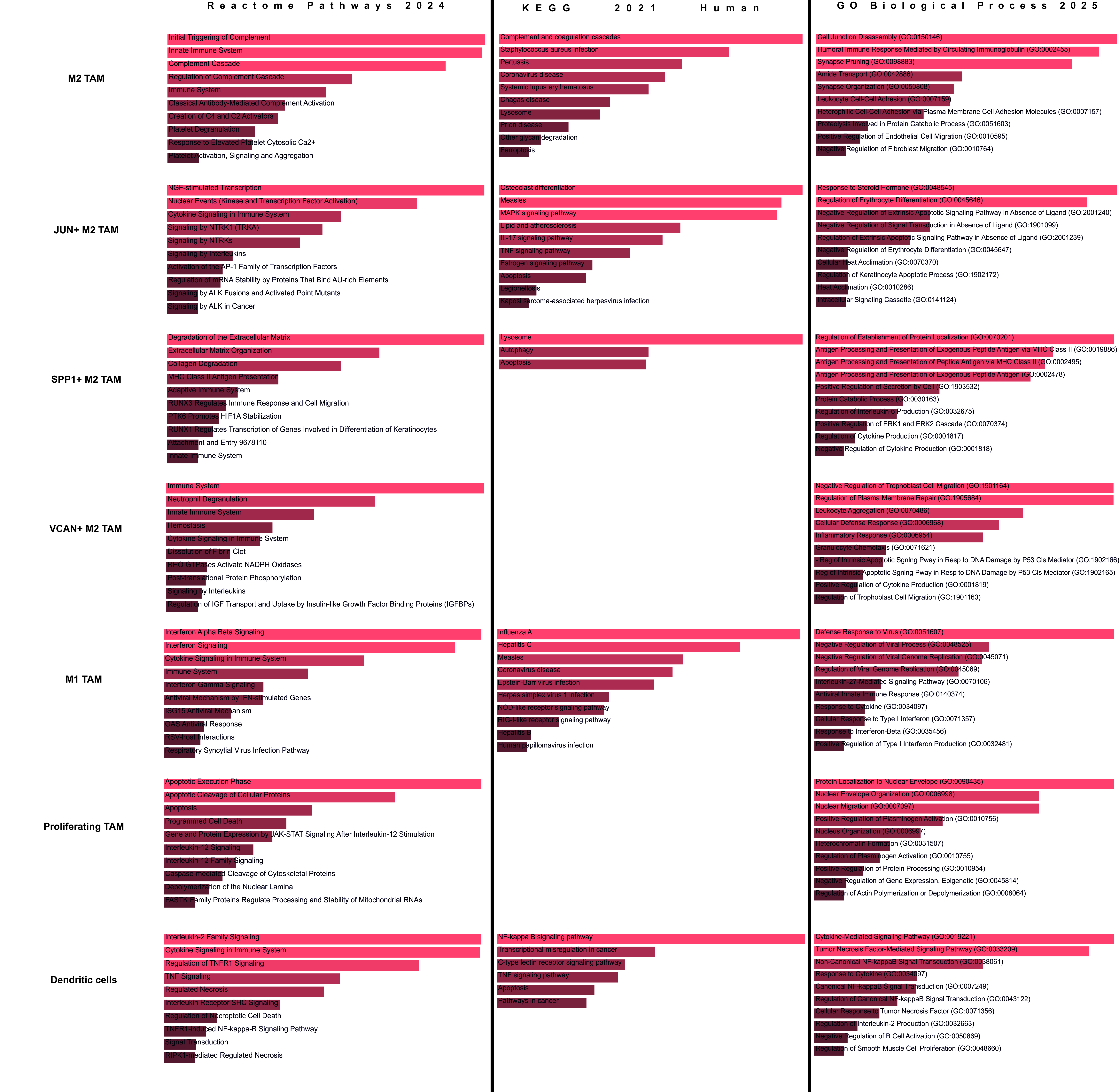

Supplement: Supplementary file 1 [file medsci-14-00077-s001.zip › Figure S2_Myeloid cell clusters.jpg]

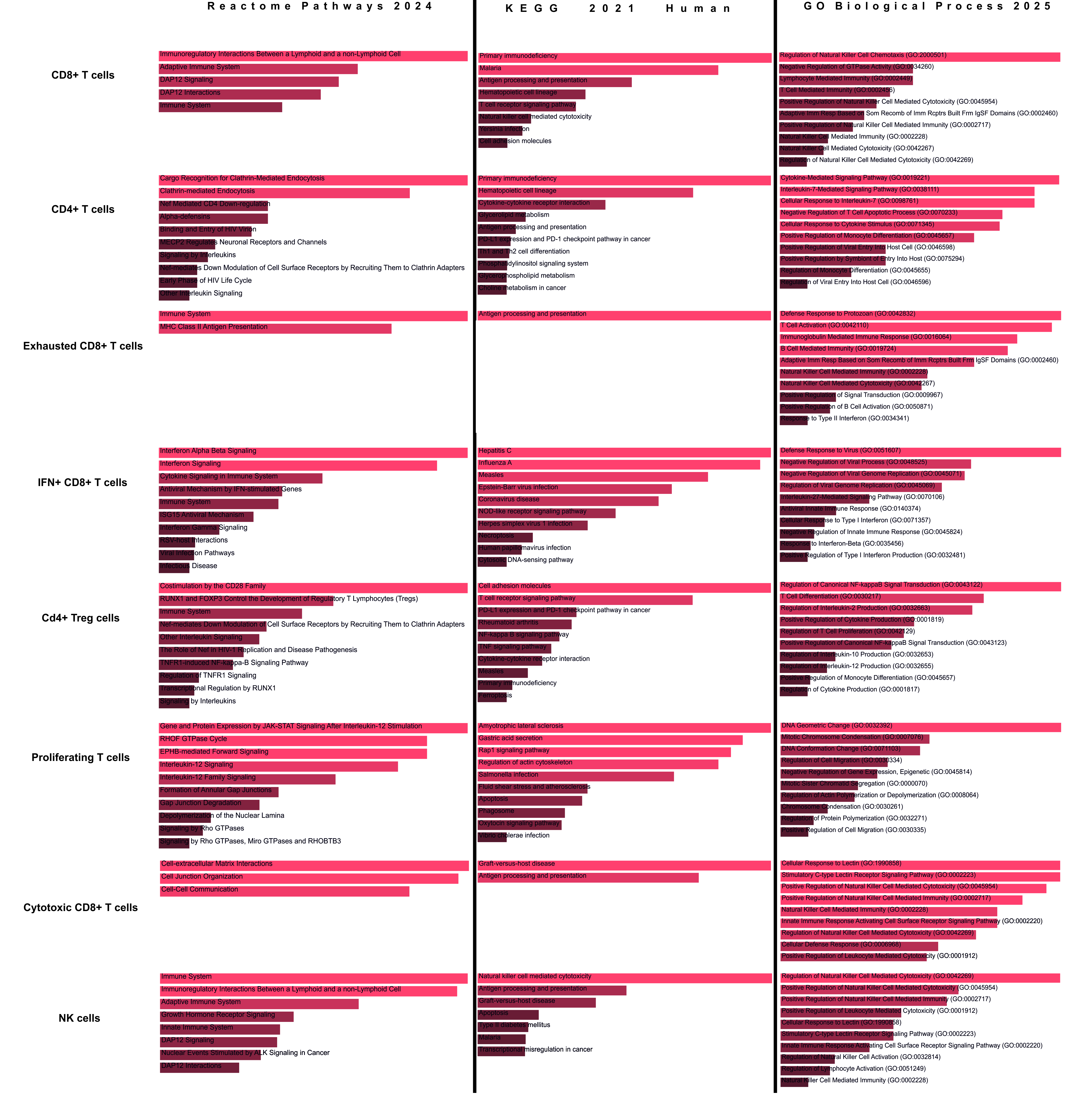

Supplement: Supplementary file 1 [file medsci-14-00077-s001.zip › Figure S3_Lymphoid cell clusters (1).jpg]

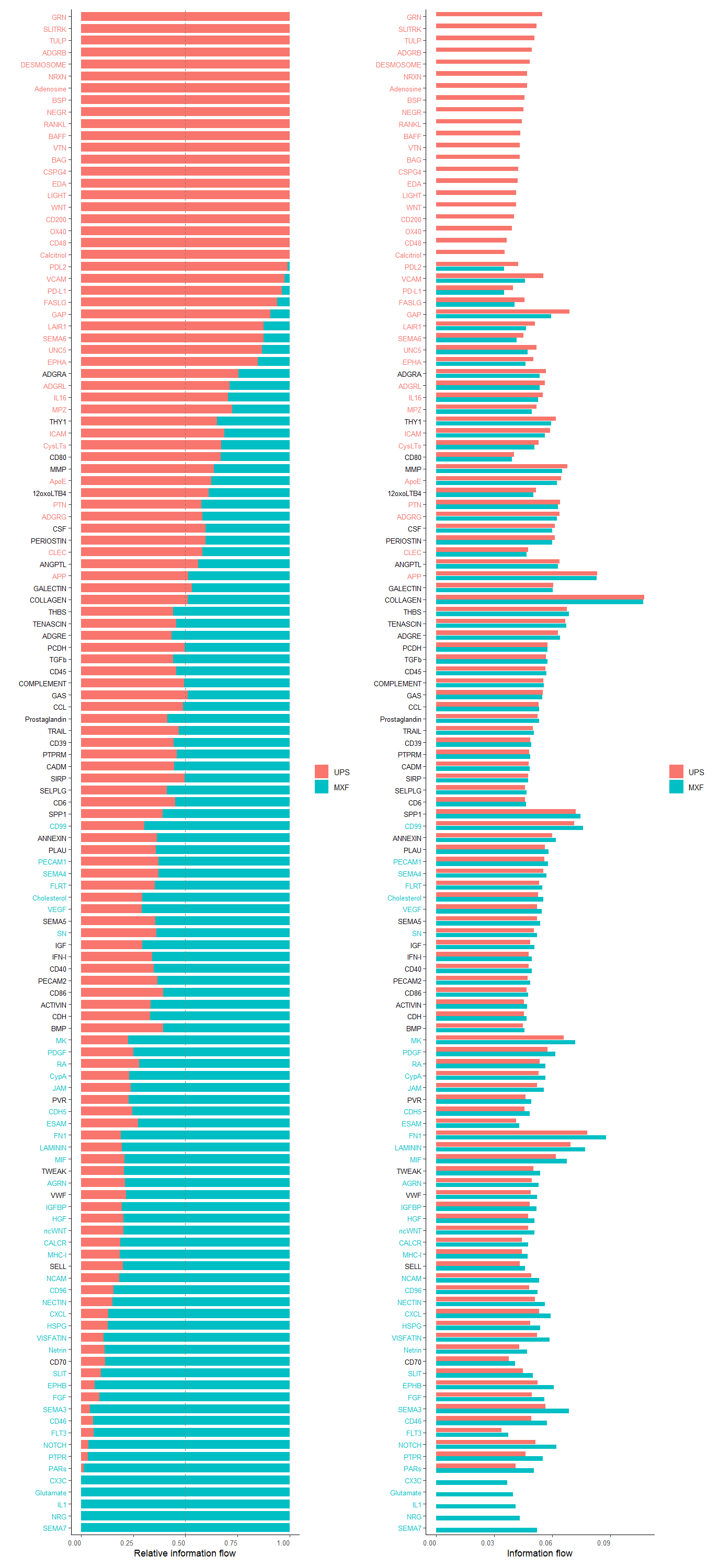

Supplement: Supplementary file 1 [file medsci-14-00077-s001.zip › Figure S4_Pathway comparison.png]
